# The acute effects of whole body vibration stimulus warm-up on skill-related physical capabilities in volleyball players

**DOI:** 10.1038/s41598-021-85158-w

**Published:** 2021-03-10

**Authors:** Chung-Cheng Wu, Min-Hsien Wang, Chi-Yao Chang, Min-Hao Hung, Hsin-Huan Wang, Ke-Chou Chen, Tzong-Rong Ger, Kuo-Chuan Lin

**Affiliations:** 1 Department of Ball Sport, National Taiwan University of Sport, Taichung, Taiwan, ROC; 2 Department of Physical Education, Chinese Culture University, Taipei, Taiwan, ROC; 3 Office of Physical Education, Chung Yuan Christian University, Taoyüan, Taiwan, ROC; 4 Graduate Institute of Sports Science, National Taiwan Sport University, Taoyüan, Taiwan, ROC; 5 Department of Biomedical Engineering, Chung Yuan Christian University, Taoyüan, Taiwan, ROC

**Keywords:** Computational biology and bioinformatics, Developmental biology, Neuroscience

## Abstract

Whole body vibration (WBV) has been suggested to improve athletes’ neuromuscular strength and power. This study investigated the effect of single WBV stimulation on volleyball-specific performance. The participants were 20 elite male volleyball players who performed a 1-min warm-up exercise on a vibration platform at a frequency of 30 Hz and peak-to-peak displacement of 2 mm. After the warm-up exercise, the participants performed a blocking agility test (BAT), 10-m sprinting test, agility T-test, and counter movement jump test. We compared the participants’ performance at four time points (Pretest, Post 0, Post 1, and Post 2). The results revealed that the participants’ BAT performance and maximum rate of force development improved significantly 1 min after the vibration stimulation (*p* < 0.01). The WBV (frequency of 30-Hz, peak-to-peak displacement of 2 mm) intervention significantly improved the volleyball-specific defensive performance and speed strength of the participants. Accordingly, by undergoing WBV as a form of warm-up exercise, the technique and physical fitness of volleyball players can be improved.

## Introduction

Recently, whole body vibration (WBV) has been applied in strength and conditioning training. Vibration platforms provide external stimulation to those engaging in strength and conditioning training through vibration with constant amplitude and frequency. Vibration platforms can be classified into two types. One type of platform generates synchronous vertical vibration, whereby vibration stimulation is transmitted to both feet simultaneously. The other type of platform generates side-alternating vibration, whereby the vibration stimulation is transmitted to both feet alternatively from left to right in the pattern of a seesaw^1^. WBV intensifies the reflex during muscle contraction, which leads to tonic vibration reflex (TVR); consequently, the proprioception of muscle spindles is affected, thereby changing the length and contraction speed of skeletal muscle fibers^2^. Studies have indicated that fixed amounts of WBV training at consistent vibration intensities stimulate muscle adaptability and improve various indices of neuromuscular function and physical fitness^3^, such as muscle strength and power^4^, flexibility^5^, body composition^6^, and delayed-onset muscular soreness^7^. Therefore, athletes can apply vibration stimulation in their training programs to improve muscle contraction. Hence, according to the aforementioned studies, WBV improves various physical fitness indices in athletes.

In addition to the effect of WBV on various physical fitness indices in athletes, recent studies^8–10^ have demonstrated using scientific models that training outcomes, professional coaching techniques and perspectives, and accurate data feedback through performance assessment instruments and methods enable athletes to improve their performance and techniques; subsequently, their individual sports performance on the court were improved. Some studies have reported that through stimulation during WBV, the leg power of athletes during counter movement jump (CMJ) tests improved^7,11^. Manimmanakorn et al.^12^ conducted a meta-analysis on the effect of WBV on jump heights and incorporated a WBV intervention in a 10-min training exercise performed for 12 weeks. Their study revealed that a vibration frequency of 30 Hz effectively improved the participants’ jumping abilities. Cormie et al.^13^ conducted a 30-s WBV leg-stimulation intervention at a frequency of 30 Hz and amplitude of 2.5 mm and explored its effect on leg muscle power; the results showed that the participants improved their CMJ heights significantly. Adams et al.^14^ examined the effect of WBV at different frequencies (30, 35, 40, and 50 Hz) and amplitudes (2 and 5 mm) on the muscle performance of participants at different time points (immediately, 1 min, 5 min, and 10 min after the intervention). After participants underwent 1-min WBV interventions in all frequency combinations and amplitudes, Adams et al.^14^ found that the muscle power of participants increased significantly immediately and 1 min after the intervention; moreover, of all applied frequencies, the frequency of 30 Hz enhanced the muscle power of participants the most. They also indicated that the effect of WBV dissipated 5 min after the intervention.

Volleyball is a team sport that involves a considerable amount of jumping, which requires satisfactory leg muscle power, during competitions. Jumping power is generated through body weight, ground reaction force, and the effective use of hip joints, ankle joints, knee joints, and leg muscles. Thus, the primary factors that affect jump performance are approach velocity, countermovement, upper body lean, arm swing, and knee extension^15^. In exploring the effect of warm-up and stretching exercises in sports medicine, Woods et al.^16^ indicated that appropriate warm-up exercise have physiological benefits (e.g., improves blood flow to muscle tissues in athletes, increases muscle contraction and neurotransmission speed, reduces the risk of sports injury, and enhances flexibility in athletes). In a study to examine the effect of acute biceps warm-up exercises, Veevo et al.^17^ reported that dynamic warm-up exercises can enhance skeletal muscle contraction in athletes and help athletes perform movements that require power. Effective warm-up exercises before training sessions or games reduce the risk of injury in volleyball players and enhance their performance. In the present study, volleyball strength and conditioning coaches were invited to discuss the effect of dynamic warm-up exercises before training and games. We investigated whether incorporating WBV into dynamic warm-up exercises improves the muscle power and sports performance of volleyball players at different time points to clarify the effect of WBV in volleyball players.

## Results

**Figure 1:**
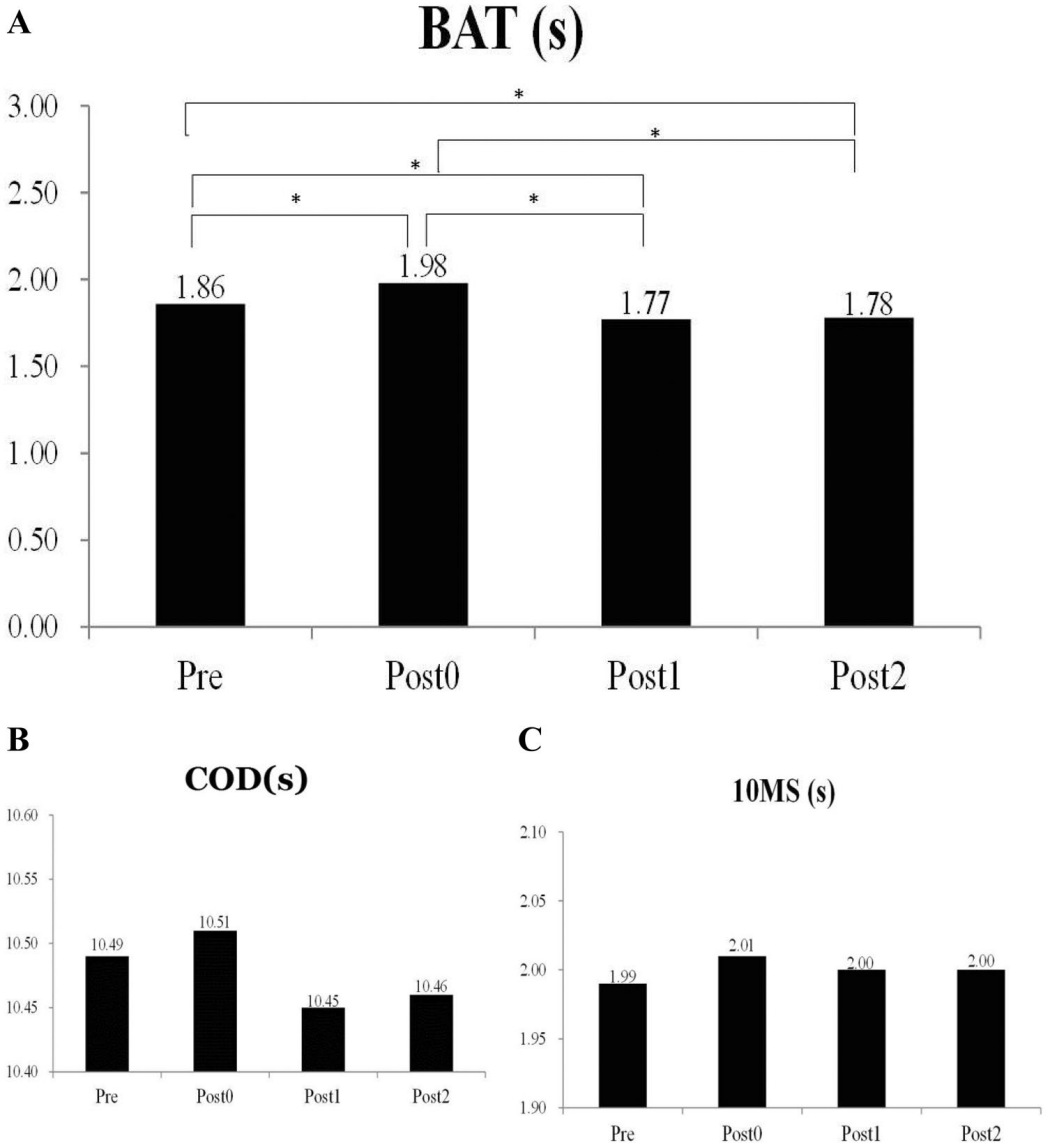
Blocking agility test (**A**) & special physical fitness (Change of direction (**B**) & 10 m sprint (**C**)) percentage difference. Data are expressed as the mean (n = 20) ± standard error. All experiments were performed in four. The statistical analysis of differences was performed using one-way analysis of variance (group × time) of variance. *p* < 0.05 was considered to indicate a statistically significant difference. Asterisk indicates significant difference compared to timeline.

**Figure 2:**
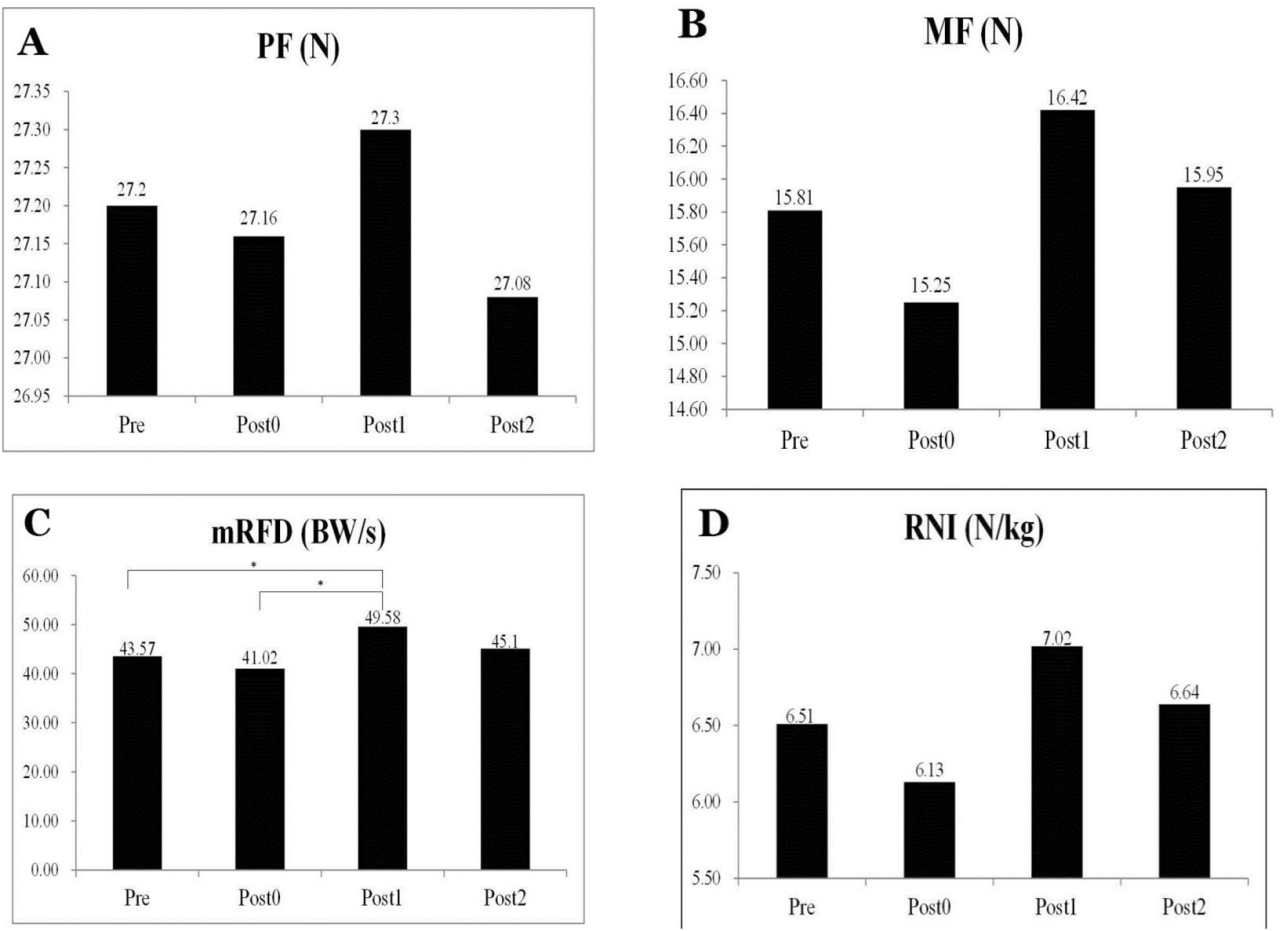
Ground reaction force percentage difference. Ground reaction force was generated when the participants jumped, and the peak force (PF) (**A**), mean force (MF) (**B**), maximum rate of force development (mRFD) (**C**), and relative net impulse (RNI) (**D**) were calculated as the indices of leg muscular force. Data are expressed as the mean (n = 20) ± standard error. All experiments were performed in four. The statistical analysis of differences was performed using one-way analysis of variance (group × time) of variance. *p* < 0.05 was considered to indicate a statistically significant difference. Asterisk indicates significant difference compared to timeline.

Descriptive statistics for agility (BAT, 10MS, COD, PF, MF, relative net impulse (RNI), and maximum rate of force development (mRFD)) are presented in Table 1. The participants’ BAT results changed significantly after the vibration stimulation (F = 28.774, *p* < 0.001); their results improved by 5.06% at Post 1 and by 4.11% at Post 2. The post hoc analysis revealed a significant difference between pretest and posttest results (Post 0: *p* = 0.001, ES = 1.25; Post 1: *p* = 0.001, ES = 1.13; Post 2: *p* = 0.012, ES = 1.13); significant differences were also observed between Post 1 and Post 0 (*p* < 0.001, ES = 2.18) and between Post 2 and Post 0 (*p* < 0.001, ES = 2.26). The BAT results are presented in Fig. 1. Figure 1 also shows that no significant differences were observed among the 10-m sprint (F = 0.218, *p* = 0.883) and COD (F = 0.149,* p* = 0.930) results, which indicated that WBV stimulation did not substantially improve participants’ performance during these tests. Regarding CMJ as the test parameter of ground reaction force, no significant improvement was noted in participants’ PF (F = 0.018, *p* = 0.997), MF (F = 0.751, *p* = 0.525), and RNI (F = 32.812, *p* = 0.280) after the stimulation. Nevertheless, WBV stimulation improved participants’ mRFD results significantly (F = 7.083, *p* < 0.001); paired comparisons revealed that their mRFD results improved significantly by 13.8% and 3.51% at Post 1 and Post 2, respectively, compared to those at the Pretest. A post hoc comparison also revealed significant differences between Post 1 and Pretest results (*p* = 0.014, ES = 0.84) and between Post 1 and Post 0 results (*p* < 0.001, ES = 1.21). Furthermore, we found a difference between the 1-min and 2-min post WBV performances during the CMJ (Fig. 2).

**Table 1:** One-way ANOVA results of the tests measured at various time points after the vibration intervention (mean ± SD).

	Pre-test (CV%)	Post 0 (CV%)	Post1 (CV%)	Post 2 (CV%)	F
BAT (s)	1.86 ± 0.08 (4.30)	1.98 ± 0.11 (5.56)	1.77 ± 0.08 (4.52)	1.78 ± 0.06 (3.37)	28.774**
10MS (s)	1.99 ± 0.08 (4.02)	2.01 ± 0.09 (4.48)	2.00 ± 0.10 (5.00)	2.00 ± 0.10 (5.00)	0.218
AT (s)	10.49 ± 0.30 (2.86)	10.51 ± 0.27 (2.57)	10.45 ± 0.26 (2.49)	10.46 ± 0.36 (3.44)	0.149
PF (N/kg)	27.20 ± 3.01 (11.07)	27.16 ± 3.23 (11.89)	27.30 ± 3.19 (11.68)	27.08 ± 3.18 (11.74)	0.018
MF (N/kg)	15.81 ± 2.55 (16.13)	15.25 ± 2.46 (16.13)	16.42 ± 2.66 (16.20)	15.95 ± 2.55 (16.99)	0.751
mRFD (BW/s)	43.57 ± 4.94 (11.34)	41.02 ± 4.77 (11.63)	49.58 ± 8.84 (17.83)	45.10 ± 5.35 (11.86)	7.083**
RNI (Ns/kg)	6.51 ± 1.51 (23.20)	6.13 ± 1.43 (23.33)	7.02 ± 1.40 (19.94)	6.64 ± 1.55 (23.34)	1.303

BW = body weight, BAT = blocking agility test, 10MS = 10 m sprint, AT = agility T-test, PF = peak force, MF = mean force, mRFD = maximum rate of force development, RNI = relative net impulse, CV = coefficient of variation.

*p < .05, **p < .01.

## Discussion

Volleyball players’ physical fitness, techniques, and physical states critically determine their likelihood of winning matches. Because of congenital factors, their physical attributes cannot be changed, but their techniques, physical states, and jumping abilities can be improved through training. Improving leg muscle strength, anaerobic exercise capacity, and muscle activation enables volleyball players to enhance their jumping abilities. In the present study, WBV stimulation was incorporated in warm-up exercises to improve participants’ sports performance. The results indicate that WBV stimulation effectively enhanced participants’ blocking agility and leg muscle power (mRFD) at Post 1 and Post 2.

In the BAT, signal lights were incorporated to facilitate visual stimulation. The participants moved two steps forward and jumped vertically to block the indicated signals within 2.5 m, during which time speed and agility were crucial for successful maneuvers. During volleyball games, blocking a ball requires satisfactory jumping ability. In the WBV warm-up exercise, the vibration platform generated vertical sine-wave vibrations, which were transmitted to the bodies of participants; the participants’ muscles, bones, and sensory organs then promptly adapted to the stimulation by activating the neuromuscular reflex. This mechanical reflex is known as TVR. When WBV stimulation is applied to activate muscles, the vibration appears to change stimulation patterns transmitted between the two ends of the muscle spindle to induce changes in the mode of neuromuscular recruitment and activation of α motor neurons. Additionally, WBV is recommended to improve the neuromuscular status of athletes; vibration applied to sensitive parts of the human body, such as the skin, joints, and secondary peripheral neural systems, has been shown to benefit γ systems and enhance the sensitivity of α motor neurons^18^. Furthermore, the TVR generated through WBV training effectively enhances athletes’ flexibility, muscle contraction speed, and nervous system performance. Studies have reported that muscle reaction time or overall muscle reaction time requires long-term training to achieve substantial improvement^19^. By contrast, manual vibration training can be completed while effectively controlling the intervention time, frequency, and physical load. It can also enhance athletes’ BAT performance within a short time.

Blocking is a basic volleyball technique that utilizes players’ physical abilities and judgement instincts. From the perspective of physical fitness, effective blocking is affected by athletes’ reactions, movement speed, and muscle power. In a volleyball match, the time available to perform the technique is considerably short. The instant effect of vibration stimulation is generated through its adaptation by the neuromuscular system. The stimulation transmitted to muscles invokes TVR, which enhances joint torque and accelerates blood flow. These are crucial factors that cause increased muscle activity. Controlling vibration frequency and amplitude is critical during the application of vibration stimulation as an external stimulus during a training intervention; the higher the load and the longer the stimulation time a participant is subjected to, the more notable its effect becomes. However, not all stimuli lead to a positive outcome.

The specific test results of participants did not improve substantially after the intervention. This may have been because the vibration frequency and amplitude were insufficient for the required muscle stimulation intensity. The warm-up energy generated through vibration stimulation is termed postactivation potentiation, which refers to the improvement of muscular properties through stimulation before activities begin. The decrease in the participants’ muscle activities in the present study was possibly caused by fatigue in their neuromuscular systems. When an external stimulus invokes muscle resistance, it delays Ca^2+^ synthesis, which affects muscle contraction speed and causes the participants to experience fatigue-like states. The postactivation potentiation caused by WBV may be related to the vibration intensity^20^. The body postures analyzed in this study were responsible for initiating muscle stretching. However, jumping involves a muscle force and action speed model different from that required during sprinting. Similarly, the WBV intervention did not significantly improve participants’ 10-m sprint and COD performance. This confirmed one of our hypotheses which was that WBV did not improve participants’ agility to change directions. Agility involves several characteristics, such as acceleration and maximum speed, which can be evaluated during a 10-m sprint test and COD, respectively. The WBV intervention implemented in this study improved participants’ rapid centrifugal and centripetal muscular power. Further study is required to investigate why the duration, intensity, and vibration model employed in the WBV intervention in this study did not enhance participants’ agility.

The CMJ test is commonly used to assess and monitor neuromuscular changes in the legs of athletes. The CMJ test reveals changes in many of the physical parameters of athletes during their sporting activities; moreover, the test can be repeated multiple times within a short period because it is safe and does not exert a high physical load. The force plate data acquired through a time domain analysis of CMJ tests can be used to evaluate the changes in the adaptation model of neuromuscular systems^21^. In the present study, no significant differences were observed in the PF and MF results at different time points; however, the mRFD and RNI results improved significantly. In other words, immediately after undergoing vibration training, the ground reaction force of participants increased during the CMJ test. Leg muscle power is the maximum neuromuscular force generated by leg muscles within a short period. CMJ tests can reveal improvements in athletes’ power output and motor neuron recruitment after they complete warm-up exercises. In volleyball, which involves extensive jumping, leg muscle power and jumping abilities critically influence athletes’ performance. In the present study, the TVR generated through the activation of α motor neurons by vibration stimulation significantly improved the mRFD performance of participants (13.8% at Post 1 and 3.51% at Post 2), which indicated an enhancement in their jumping abilities. Therefore, vibration training should be able to effectively improve athletes’ muscular performance.

No intervention-based assessment has been conducted on volleyball-specific actions in related studies on vibration stimulation. In the present study, tests were conducted to assess the participants’ reaction time during volleyball defense actions to explore the effect of WBV stimulation on volleyball athletes’ specific actions and blocking performance. The results revealed that WBV is applicable as a warm-up exercise for volleyball players before specific training sessions or games. WBV stimulation was confirmed to improve volleyball players’ blocking agility by enhancing mRFD and faster approaching movement.

In conclusion, the BAT, RNI, and mRFD power can be significantly improved through a single session of WBV training lasting for 1 to 2 min. Such an intervention can help the volleyball players to complete dig movements rapidly in competitions. By using quantitative methods, the physical and psychological stability of athletes can be monitored. All these methods can be used to help players improve their physical abilities and skills level. The results of this study are similar to those reported in past studies. We showed that blocking agility was effectively improved by WBV training. Additionally, the greater the RNI and mRFD, the more rapidly and effectively defense movements were completed.

## Methods

### Experimental approach to the problem

This study examined the effect of single WBV stimulation training as a warm-up exercise on volleyball players’ specific performance. The duration of the study designs without inference 5–15 min. We used the findings of previous studies as reference^8^. Previous studies showed that after the WBV training, it will take five minutes to return to the initial state. Additionally, the games-to-games match within an average of 3 min. The participants were 20 male university or college volleyball players, all of whom were first-division players in the University Volleyball League from the level 1 Team. All the participants had undergone professional volleyball training for at least five years, and none had participated in neuromuscular mechanical stimulation training within the six months before the study; avoidance of any previous stimulation-induced muscle activation methods, leading to neural adaptations to enhance muscle strength. Approval from the relevant local Institutional Review Board (Landseed International Hospital Institutional Review Board [18–015-B1]) and individual written informed consent from all participants were obtained beforehand. All experiments were performed in accordance with relevant local guidelines and regulations.

### Experimental design

The tests involved in this study were the blocking agility test (BAT), 10-m sprinting (10 m-sprint) test, change of direction test (COD), and CMJ test. To prevent muscle activation and fatigue, the data were collected at 48-h intervals for each test. Because the effects of WBV are extremely time sensitive^13^, the BAT, 10 m-sprint, COD, and CMJ data were collected at Post-0, one minute (Post 1), and two minutes (Post 2) after each WBV bout.

### Procedures

#### WBV stimulation

All participants stepped on a vibration platform (AV-009, Body Green, Taipei, Taiwan) to receive synchronous vertical mechanical vibration stimulation. The vibration stimulation, delivered at a frequency of 30 Hz and an amplitude of 2 mm for 1 min, was based on previously conducted training interventions that effectively improved users’ muscle power and strength^13^. The participants bent their knees at an angle of approximately 120°, which was verified by Cormie et al.^13^ as the angle that most effectively enhanced the physical performance of athletes. The participants wore volleyball shoes to prevent injury, and the vibration absorption capacity of each pair of shoes was standardized among the participants. A pretest was conducted to obtain the baseline performance of participants before the vibration intervention began (Pretest); post tests were performed immediately (Post 0), 1 min (Post 1), and 2 min (Post 2) after the intervention. The participants were informed of the test procedures and underwent a nonstandard training session as a familiarity test before the experiment began. All the tests involved the same sports performance and physical fitness tests and stimulation intervention procedures.

### Testing procedures

#### Blocking agility test (BAT)

This test was conducted by using a visual stimulation agility system to evaluate each participant’s blocking reaction capability. Random visual stimulations were provided in the form of signal lights, and the blocking total reaction times performed to touch the signals were recorded. The BAT sampling frequency was set at 1000 Hz. A computer was used for instant data display and storage^22^. In reference to the methods employed by Sheppard et al.^23^ the blocking target was placed 2.5 m away from the starting position of participants, and the blocking height was set at 90% of each participant’s personal jump height. Each participant performed a blocking move 14 times; each block was performed at 8-s intervals^24^.

#### Special physical fitness

1. 10-m sprint (10 m-sprint)
  Interval timers were used during the test for data recording. A timer was placed at the start and finish lines. The heights of timers were set to that of each participant’s hip to prevent scoring errors caused by hip swinging. Each participant began the test in a standing position (split stance). Each participant in a standing position with the toe of the preferred foot forward 0.3 m behind the timer gate. This was intended to allow some forward lean and to provide triggering of the timer as soon as the subject moved. An infrared photoelectric circuit breaker timer was employed to record the time the participant crosses the starting line. The recording process stopped when the timers detected that the participant had crossed the finish line. The test was conducted three times for each participant, and the average performance of all the three 10 m-sprint tests was calculated as the final 10 m-sprint performance of each participant.
2. Change of direction (COD)

This test was based on the COD conducted by Miller et al.^25^ Each participant ran for a total distance of 36.56 m. The processes involved when changing running directions were used to evaluate participants’ agility. Each participant started at the first traffic cone (Cone A) and ran for 9.14 m. The participant then touched the second cone (Cone B) with his right hand, moved left for 4.57 m while facing forward, and touched the third cone (Cone C) using his left hand. The participant then moved right for 9.14 m while still facing forward and touched the fourth cone (Cone D) using his right hand. Subsequently, the participant moved left for 4.57 m while facing forward and touched Cone B again before running backwards to Cone A. The test was intended to evaluate the speed at which participants changed directions; interval timers were used for data recording. Each participant performed the test three times, and the average performance was calculated as the final COD performance of participants. The sampling frequency of the interval timers was set to 200 Hz.

#### Ground reaction force parameter test of lower limbs

The muscle force of participants’ legs was examined according to their CMJ performance on a force plate (9260AA, Kistler Ltd., Switzerland). Ground reaction force was generated when the participants jumped, and the peak force (PF), mean force (MF), maximum rate of force development (mRFD), and relative net impulse (RNI) were calculated as the indices of leg muscle force according to the relevant methods defined by Gathercole, Sporer, Stellingwerff, and Sleivert^21^. Each participant performed three jumps, and the mean of all jump heights was calculated as the CMJ result for each participant. The data were standardized according to the body weight of each participant. The sampling frequency of the force plate was set as 1000 Hz.

## Data Availability

Data from all the tests (BAT, 10 m-sprint, COD, and CMJ test) were processed using a custom-written MATLAB script(Version R2008a; MathWorks Inc., USA). The statistical differences among the Pretest, Post 0, Post 1, and Post 2 results were analyzed using SPSS (Version 20.0; SPSS Inc., Chicago, IL, USA). Repeated measures one-way analysis of variance (time) and the Bonferroni post hoc method were employed to evaluate participants’ BAT, 10 m-sprint, COD, PF, MF, mRFD, and RNI results at different time points after the vibration stimulation. The level of significance was set at *α* = *0.05.*
